# A longitudinal electrophysiological and behavior dataset for PD rat in response to deep brain stimulation

**DOI:** 10.1038/s41597-024-03356-3

**Published:** 2024-05-15

**Authors:** Xiaofeng Wang, Min Chen, Yin Shen, Yuming Li, Shengjie Li, Yuanhao Xu, Yu Liu, Fei Su, Tao Xin

**Affiliations:** 1 Department of Neurosurgery, The First Affiliated Hospital of Shandong First Medical University & Shandong Provincial Qianfoshan Hospital, Jinan, 250014 China; 2 Medical Science and Technology Innovation Center, Shandong First Medical University & Shandong Academy of Medical Sciences, Jinan, 250117 China; 3 Department of Radiology, Shandong First Medical University & Shandong Academy of Medical Sciences, Taian, 271016 China; 4 Centre for Biosystems, Neuroscience, and Nanotechnology, City University of Hong Kong, Hong Kong, 999077 China; 5 Division of Nuclear Technology and Applications, Institute of High Energy Physics, Chinese Academy of Sciences, Beijing, 100049 China; 6 Department of Neurology, The Second Affiliated Hospital of Shandong First Medical University, Taian, China; 7 Shandong Institute of Brain Science and Brain-inspired Research, Shandong First Medical University & Shandong Academy of Medical Sciences, Jinan, 250117 China

**Keywords:** Neuronal physiology, Regeneration and repair in the nervous system

## Abstract

Here we presented an electrophysiological dataset collected from layer V of the primary motor cortex (M1) and the corresponding behavior dataset from normal and hemi-parkinson rats over 5 consecutive weeks. The electrophysiological dataset was constituted by the raw wideband signal, neuronal spikes, and local field potential (LFP) signal. The open-field test was done and recorded to evaluate the behavior variation of rats among the entire experimental cycle. We conducted technical validation of this dataset through sorting the spike data to form action potential waveforms and analyzing the spectral power of LFP data, then based on these findings a closed-loop DBS protocol was developed by the oscillation activity response of M1 LFP signal. Additionally, this protocol was applied to the hemi-parkinson rat for five consecutive days while simultaneously recording the electrophysiological data. This dataset is currently the only publicly available dataset that includes longitudinal closed-loop DBS recordings, which can be utilized to investigate variations of neuronal activity within the M1 following long-term closed-loop DBS, and explore additional reliable biomarkers.

## Background & Summary

Deep brain stimulation (DBS) is a complementary treatment to drug therapy, which is important for the treatment of movement disorders, such as advanced drug-refractory Parkinson’s disease (PD), dystonia, and essential tremor^1,2^. The last 30 years have witnessed the rapid development of DBS, and shown significant improvement in symptoms such as tremor, and bradykinesia in PD patients, however, there is still a lack of understanding of its mechanism. Some studies prove that DBS achieves its symptomatic treatment by regulating the neuronal oscillation activity through the application of high frequency stimulation to specific nuclei^3^. The generation, coding, transmission and integration of information in the brain are completed through the action potentials of neurons, because neuron is the smallest structure and functional unit of the nervous system^4–6^. The recording of individual neuron activity and local neuron population activity could help obtain the characteristics of action potentials, and also decode brain activity from multiple scales^7–9^, which is particularly important for exploring the mechanisms of DBS^10,11^.

Studies in animal models of PD have reported that DBS applied in the basal ganglia nuclei can also modulate cortical activity^12–14^. This suggests that DBS may act through multiple synergetic mechanisms^15^, rather than simply inhibiting or exciting the basal ganglia nuclei. Dopamine depletion is accompanied by elevated beta-band oscillatory activity in the cortico-basal ganglia-thalamic motor control loop^16–20^. Although the mechanisms leading to the abnormal beta-band oscillatory activity are still unclear, the abnormal oscillatory activity also indicate that significant changes occur in the neural network and neuronal information transmission of the cortico-basal ganglia-thalamic loop. Decoding the characteristics of these abnormal activities could enhance our understanding of how they affect the cortico-basal ganglia-thalamic motor loop, facilitate development of new therapeutic approaches, and also help explore the potential mechanisms of DBS.

DBS provides a unique opportunity to study the neurophysiological activity of PD *in vivo*, the intraoperative microelectrode recording is mainly used to determine the anatomical structure of the stimulation target and improve localization accuracy^3^, however, the long-term neural activity information cannot be obtained. The newly emerging sensing DBS devices like Percept PC offer a new opportunity to continuously record the local field potentials (LFPs) from the implanted DBS leads^21,22^, unfortunately, they cannot collect neural activities beyond the stimulation target region. DBS intervention is a long-term and chronic process for PD patients, however, current studies mainly focus on the acute effects of DBS^10,23,24^, with stimulation time varies from minutes to hours, lacking information of neural activity under long-term effective stimulation. Brain network dynamics endow the brain with a huge potential of neuroplasticity^25^, long-term repetitive stimulation can lead to the variation of neural plasticity^26^. DBS has been shown to effectively improve the gait and posture of PD patients, with neuronal plasticity potentially playing a crucial role in this process^27^, at the same time, there are accompanying neurochemical changes^28^, thus the neurobiological response to acute stimulation may not represent changes caused by chronic stimulation.

Further studies are necessary to explore the effects of long-term DBS intervention on the neurophysiology, neurotransmitters, and functional metabolism of the cortico-basal ganglia-thalamic motor loop. This is because the changes induced by long-term DBS may better reflect variations in the brain network and fluctuations of movement states^29^. Decoding neurophysiological information based on long-term DBS, and utilizing such signals as biomarkers of clinical symptom variations or disease severity can guide the adjustments of stimulation parameters, and finally form the closed-loop DBS system^30^. This will help further investigate the therapeutic mechanism of DBS, minimize side effects caused by continuous stimulation, and enable individualized stimulation in future applications.

Rodents have always been valuable tools for studying human diseases. Although existing animal models cannot fully replicate clinical conditions, models constructed through neurotoxin injections or gene knockout techniques have successfully mimicked behaviors observed in PD patients^31^. By stereotactically injecting a specific catecholaminergic neurotoxin 6-hydroxydopamine (6-OHDA) into the medial forebrain bundle (MFB), the hemi-parkinsonian (hemi-PD) rat model can be constructed, which exhibits abnormal beta band oscillatory activities recorded within motor control involved regions, e.g. the primary motor cortex (M1), subthalamic nucleus (STN), and the substantia nigra pars reticulate (SNpr)^32,33^. Although the beta-band frequency range of hemi-PD rat (12–40 Hz) is slightly higher than PD patients(12–30 Hz)^34^, the similarity also provides an excellent opportunity to study mechanisms behind the significantly elevated beta-band oscillatory activity and its modulation through DBS intervention.

In this study, the microwire array electrode was implanted in layer V of M1 for normal and hemi-PD rats to perform longitudinal recording. The multi-unit spike signals and neuronal population LFPs were analyzed to evaluate the evolution of PD pathology. The comparisons of data among sleep, wake, and walk states were done to explore features directly correlated with different PD physiological states. On this basis we constructed a closed-loop DBS protocol for hemi-PD rats, where the DBS was turned on/off based on the oscillation activity of M1-LFP signal. The corresponding electrophysiology data was recorded for five consecutive days, which provided a unique opportunity to analyze the longitudinal electrophysiological variation of M1 after long-term closed-loop DBS and further explore more potential biomarkers.

## Methods

All animal procedures were conducted in conformity with relevant institutional rules in compliance with the National Institutes of Health Guide for Care and Use of Laboratory Animals. Protocols were approved by the Animal Ethics Committee of the First Affiliated Hospital of Shandong First Medical University (Approval No.2021080702). Efforts were made to minimize pain and the number of animals used during the experiment. The Sprague-Dawley rats (female, 180–260 g, Jinan Pengyue Experimental Animal Breeding Co., Ltd) used were housed in standard SPF animal rooms, 3 rats per cage, under a 12 h/12 h light-dark cycle, and were provided with food and water ad libitum.

The behavioral and electrophysiological data were continuously collected from the rats at different time periods, and the acquisition process was performed in a quiet test room. The open-field test was conducted and recorded to evaluate the behavior variation of rats. The OmniPlex Neural Recording Data Acquisition System (Plexon Inc., Dallas, Texas, USA) was used to record the wideband electrophysiological data, where rats were placed in a tailored Faraday cage with free access to food and water.

### 6-OHDA lesion experiment

All rats underwent at least 7 days of handling in order to familiarize them with the experimenter and the test apparatus prior to the surgery. Rats were anesthetized with isoflurane (Shenzhen RWD Life Science Co., Ltd) inhalation in a closed anesthesia induction box. During the surgery, low-flow was maintained by a small animal anesthesia machine (Beijing Zhongshi Dichuang Technology Development Co., Ltd), when the corneal and nociceptive reflexes disappeared the anesthesia was fully effective. The rats were administered desipramine (5 mg/kg, SigmaAldrich) and pargyline (50 mg/kg, SigmaAldrich) to inhibit monoamine oxidase and protect noradrenergic neurons^35^. Both eyes were covered with saline cotton balls to prevent corneal dehydration, the OxyBuprocaine Hydrochloride Gel (shenyang lvzhou pharmaceutical Co., Ltd) was applied to reduce ear discomfort, and a thermostatic heating pad was used to continuously maintain body temperature at 37°C. The rat was fixed on a stereotaxic frame (Beijing Zhongshi Dichuang Technology Development Co., Ltd), and the coordinates of drug injection and electrode implantation were determined by the rat brain atlas^36^. The hair between the line of two eyes and the line of two ears was removed, the skin was cut and the subcutaneous tissue was separated after disinfection. The periosteum on the skull surface was removed, the MFB (AP −4.4 mm, ML −1.2 mm, DV −7.8 mm) was located, and a small hole on the skull surface was opened with a tooth drill.

In this study 36 rats were used, and 10 rats were randomly selected as the sham group, while another 26 rats were selected as the PD group. For the sham group, 4 μl of 0.02% ascorbate saline solution (SigmaAldrich) was slowly infused into the left MFB area via microsyringe. For the PD group, the 6-OHDA (4 μl, 5 μg/μl in 0.2% ascorbic acid dissolved with 0.9% saline, SigmaAldrich) was infused into the left MFB to produce hemi-PD model, and meloxicam (SigmaAldrich) was given after the surgery to relieve pain. One week after 6-OHDA was lesioned, apomorphine-induced rotational analysis was used to verify the degree of dopamine depletion, with subcutaneous injection of apomorphine (0.5 mg/kg, SigmaAldrich)^37^. The hemi-PD rat model was considered successful with net contralateral rotations over 210 turns after 30 min apomorphin injection^10^. According to this criteria, 23 rats were regarded as the hemi-PD rat model, and further randomly divided as the PD group (n = 10) and PD-DBS group (n = 13).

### Design of the microwire array electrode

In this study, the stimulation and recording electrodes were all microwire array electrodes. To ensure long term stability and high-quality signal acquisition, the electrode parameters were specially customized, and the nichrome wire with high impedance, low noise and high mechanical strength was selected for recording electrodes. The recording microwire array was arranged in 4 × 4 array (nickel-titanium, 25 μm diameter, 300 μm interelectrode spacing, 8 mm length, Suzhou Kedou Brain Computer Technology Co., Ltd), and two silver wires with 125 μm diameter were reserved at the electrode connector as ground wires. The stimulation microwire array was arranged in 2 × 2 array (tungsten, 50 μm diameter, 250 μm interelectrode spacing, 10 mm length, Beijing Plexon Co., Ltd). Only 2 mm of electrode wire tips of the recording and stimulation electrodes were exposed, and the rest were encapsulated and fixed with polyethylene glycol. The recording and stimulation electrode wires were covered with a 3 μm parylene insulating layer to further improve the biocompatibility of the electrode.

### Implantation of the microwire array electrode

The stimulation electrode was implanted in the left STN (AP −3.6 mm, ML −2.6 mm, DV −7.6 mm) for the sham group (n = 10), PD group (n = 10) and PD-DBS group (n = 13), and the recording electrode was implanted bilaterally in layer V of the M1 (AP 2.5 mm,ML ± 3 mm,DV −1.6 mm) for the PD group and PD-DBS group. Since the 6-OHDA lesion was performed unilaterally, the contralateral hemisphere of the PD group and PD-DBS group could serve as its own control, so for the sham group the recording electrode was implanted only in the left M1 area. 4 stainless steel screws were implanted in rats on the surface of the skull for winding and fixing the ground wire. The electrodes were fixed on the surface of the skull with dental acrylic and screws. Penicillin and meloxicam were injected intramuscularly for 3 consecutive days after operation to prevent infection and alleviate pain.

### The open-field behavioral assessment

**Fig. 1:**
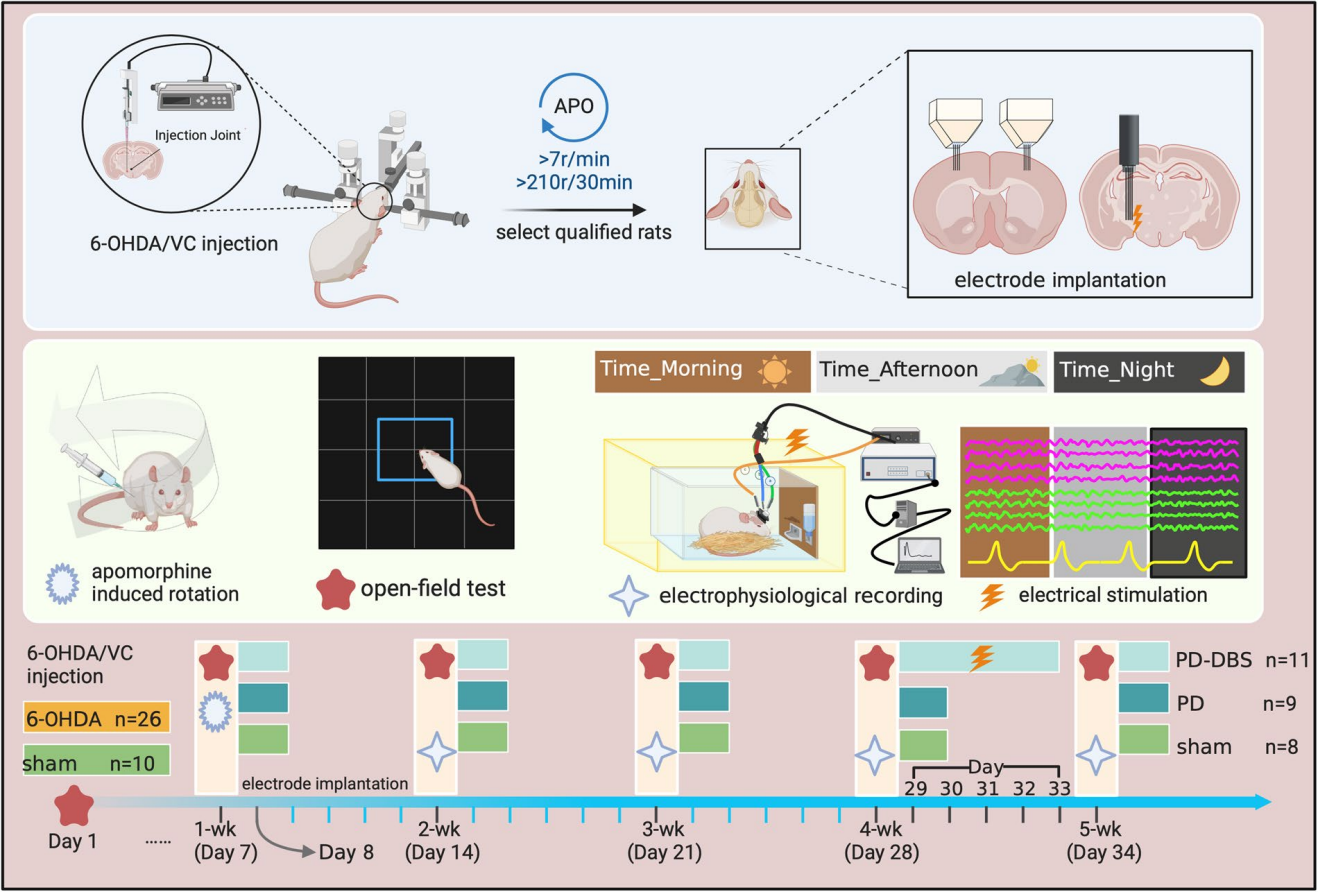
The whole experimental design. The 6-OHDA or ascorbic acid was injected into the MFB on day 1. After 6 days, the rats were further determined as hemi-PD rat model by apomorphine induced rotation. The open-field behavior test was assessed in all rats on days 7, 14, 21, 28, and 34. The stimulation and recording electrodes were implanted on day 8. For each recording, we provided electrophysiological data at different activity states in three time periods, i.e., in the morning, afternoon and night. DBS stimulation was applied to the hemi-PD rat from day 29 to day 33.

To avoid activity differences caused by circadian rhythms, the behavioral test was kept at the same time period every week, and the rats were acclimated to the surroundings in the test room 30 min before the test. The transfer of experimental rat was done through small feeding cages, shortening the time to grasp the animals as much as possible, thus to reduce the fearful stress reactions caused by moving the rats. The experimental apparatus was cleaned with clean water and 75% alcohol to remove the feces and odor left by the previous rat before and after each test, and conduct the next rat experiment after the apparatus was thoroughly dried. The behavioral test results of the rats before operation were used as the baseline data, and in the follow-up experiment, the behavioral test was done only for the sham group, PD group and PD-DBS group. The schedule of behavioral test is shown in Fig. 1, all tests and data analysis were done by two experimenters who were not aware of the grouping.

The open-field test was conducted to evaluate the general behavior and locomotor activity of rats in a new environment. The experimental device was constituted by a 100 cm × 100 cm bottom black acrylic board, and a wall surrounded by 40 cm height white acrylic board with an aluminum alloy frame. The test was conducted in a quiet, dimly lit room. The rats were gently placed at the center of the device and allowed to freely explore the device for 5 min before test. The movement was recorded by a high-definition camera above the device, the amount and duration of movement were calculated using Tracking Master 3.0 video behavior analysis software (Shanghai fanbi Smart Technology Co., Ltd). Repeated tests may cause the rats to form habits, and thus result in reduced behavioral flexibility^38^. The open-field tests were performed with one-week interval as current studies^39,40^ to more truly reflect the rats’ behavior variations among different groups.

### DBS stimulation parameters setting and electrophysiological data recording

The effect of commercial DBS system heavily depends on the parameters of the applied stimulation pulses, including the waveform, pulse width, frequency, and amplitude^41,42^. For the waveform, the monophasic pulses may generate toxic products by electrochemical reactions^43^, while charge-balanced biphasic pulses may prevent irreversible chemical reactions in the brain tissue around the electrode contacts^44,45^ and are generally used in clinical DBS for safety. For the stimulation frequency, in comparison with low-frequency stimulation^46,47^, the high-frequency (>100 Hz) stimulation is an established medical treatment for PD motor symptoms^48^. However, high-frequency stimulation may cause attention and cognitive impairment, potentially resulting from the long-term continuous stimulation.

Notably, the human subthalamic nucleus (STN) is 300 times larger than that of the rat^49^, and due to this huge difference in volume, using the same stimulation current intensity (amplitude) as in humans may result in the current spread to a larger area of the brain tissue and affect the function of other nuclei^1,50^. Thus, the current intensity was selected according to the response of each rat. Specifically, when the stimulation was turned on, the rat responded with slight involuntary movements, manifested as contralateral orofacial twitches^51,52^, then with further increase of the stimulation current intensity, sever motor impairment of the contralateral forelimb or even contralateral rotation could occur^53^. Thus for each rat, the stimulation frequency was selected as 130 Hz, the pulse duration as 90 μs, the initial current intensity was set as 20 μA, with an increase of 5 μA each time. When contralateral orofacial twitches were observed, the corresponding current intensity was set as the threshold, and in subsequent stimulation, the intensity was set 5μA lower than this threshold over 5 consecutive days.

For the PD-DBS group rats, the closed-loop STN-DBS was applied for 5 consecutive days. The closed-loop DBS control model was developed by integrating the power analysis of each frequency band in the left M1-LFP signal with the random forest algorithm, enabling real-time evaluation of rat status. For the random forest algorithm, the number of trees was set as 200, and the maximum depth of the tree was set as default (odes are expanded until all leaves are pure). Since the high beta band oscillation activity was not obvious during the non-REM sleep state^54^, simply using the beta band oscillation power as the feedback signal for a long time cannot realize accurate stimulation of rats all day long. Thus, we extracted frequency bands from 2–50 Hz, with the initial frequency band being 2–10 Hz, and the moving size was 4 Hz, i.e., a total of 11 frequency bands. These 11 frequency bands were used as the feature set of the random forest model to construct a model that can decode the PD state. We trained a personalized model for each rat, when the model identified the rat status as PD, the stimulation with well-chosen parameters was turned on, conversely, when normal power performance was observed across all frequency bands, the stimulation was turned off.

**Fig. 2:**
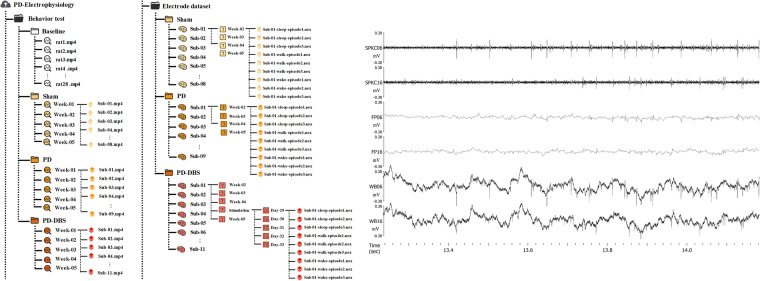
The structure and form of the dataset. (**a**) The storage form of all electrophysiological data, each data group (sham, PD, PD-DBS) contains subfolders divided by the recording time. In each recording time period, channel 1 to channel 16 were the data of lesioned side, and channel 33 to channel 48 were the data of contralateral side. (**b**) Examples of data form in each subfolder, including the LFP, wide band, and spike signals from top to bottom.

The electrophysiological data of M1 under closed-loop DBS was simultaneously recorded. The OmniPlex Neural Recording Data Acquisition System could filter the neural signals of spike and LFP from the wideband signal, which were selected with band-pass filter set at 300–3000 Hz and 0.5–300 Hz, respectively, and further digitized at a sampling rate of 40 kHz and 1 kHz. The wideband signal, spike, and LFP data files were all saved for further offline analysis (shown in Fig. 2).

Through the above setting of stimulation parameters and closed-loop modelling, the study of rodent DBS could provide valuable references for the preclinical studies of DBS in non-human primates and humans.

### Electrode location and immunohistochemistry verification

Following completion of experiments, all rats were euthanized with isoflurane, the heart was exposed through thoracotomy and then perfused transcardially with saline followed by 4% paraformaldehyde (PFA, 0.1 M, pH = 7.4, Wuhan Servicebio Technology Co., Ltd). Brains were postfixed with 4% PFA, then after dehydration, embedding and slicing for subsequent staining. Nissl staining was performed to determine the electrode position, only electrophysiological data of rats with correctly electrode implantation was used in subsequent analysis. The pathological slices of striatum and the compact part of substantia nigra (SNc) were stained by tyrosine hydroxylase (TH, ab137869, abcam) immunohistochemistry to better assess the loss of dopaminergic neuron. The histopathological changes of the striatum and SNc were visualized with an optical microscope (Olympus Optical Co. Ltd., Tokyo, Japan) at 400 × magnification.

## Data Records

The dataset was released in the G-Node format and can be downloaded at 10.12751/g-node.lzvqb5^55^. The README.md file describes the detailed information about the repository content and structure. The data of 8 rats in sham group, 9 rats in PD group and 11 rats in PD-DBS group were collected throughout the study. The ‘baseline’ folder contains data from 28 rats, ‘sham’ folder contains data from 8 rats, the ‘PD’ folder contains data from 9 rats, and ‘PD-DBS’ folder contains data from 11 rats.

We stored all the electrophysiology data in folder ‘electrode dataset for hemi-parkinsonian rat’, which contained three different groups (PD, PD-DBS, Sham). The data collected under different activity states were divided into episode 1, episode 2, and episode 3 corresponding to different collection times, i.e., in the morning, afternoon, and evening. For example, ‘sham / sub-01 / week-02 / sub-01-wake-episode1.nex’ was the electrophysiological data of the first rat in the sham group recorded during the wake state at the second week in the morning, where the ‘.nex’ file contained the raw wideband signal, neuronal spikes, and LFP signal. The ‘PD’ subfolder contained data with similar meaning to the ‘sham’ folder. The ‘PD-DBS’ subfolder not only included data of four consecutive weeks, but also the electrophysiological data of closed-loop DBS from day 29 to day 33. For example, ‘PD-DBS/ sub-05 / stimulation / Day31 / sub-05-sleep-episode1.nex’ was the electrophysiological data of the fifth rat in PD-DBS group under closed-loop DBS during sleep state on day 31 in the morning.

All behavior test data was stored in the ‘behavior test’ folder, including three subfolders of different groups (PD, PD-DBS, and Sham) and the baseline open-filed data. For example, ‘behavior test / sham / week-02 /sub-01’ was the video data recorded by a high-definition camera during the open-field test in the second week for rat 1.

Finally, we provided Python code named ‘artifacts_removal.py’ that can be used to obtain the clean LFP signal from the wideband signal, as well as their power spectral analysis to ease the reproducibility of these demonstrations.

## Technical Validation

### Histologic verification

**Fig. 3:**
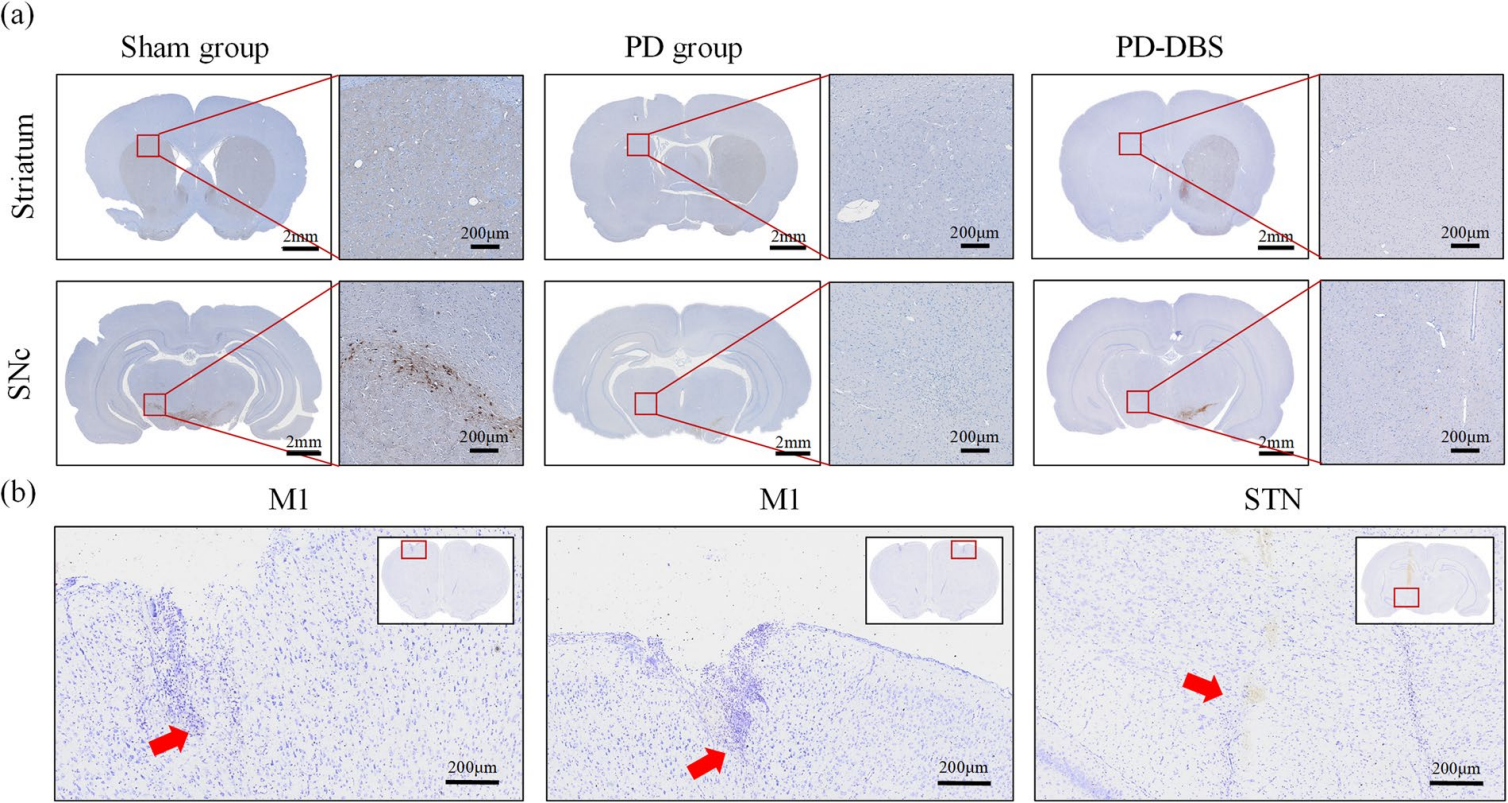
Histologic verification. (**a**) The TH-stained representative coronal section of striatum and SNc for both sham and hemi-PD rats. There was no significant difference of TH^+^ neurons between ipsilateral and contralateral sides in the sham group, however no TH^+^ neurons of ipsilateral side were found in PD and PD-DBS groups. (**b**) The position of electrode tips (red arrows) in the M1 and STN was shown by Nissl staining.

The unilateral injection of 6-OHDA produced unilateral loss of nearly all TH^+^ dopamine neurons in the striatum and SNc, however, no obvious difference was observed in the sham rat group (Fig. 3a). The location of implanted electrodes was confirmed by Nissl staining (Fig. 3b), only rats with recording microwire array electrode residing within the layer V of M1 and stimulation electrode within the STN were selected for further electrophysiological data analysis, where five rats (sham group n = 2, PD group n = 1, PD-DBS group n = 2) were excluded due to error location.

### Behavior verification

**Fig. 4:**
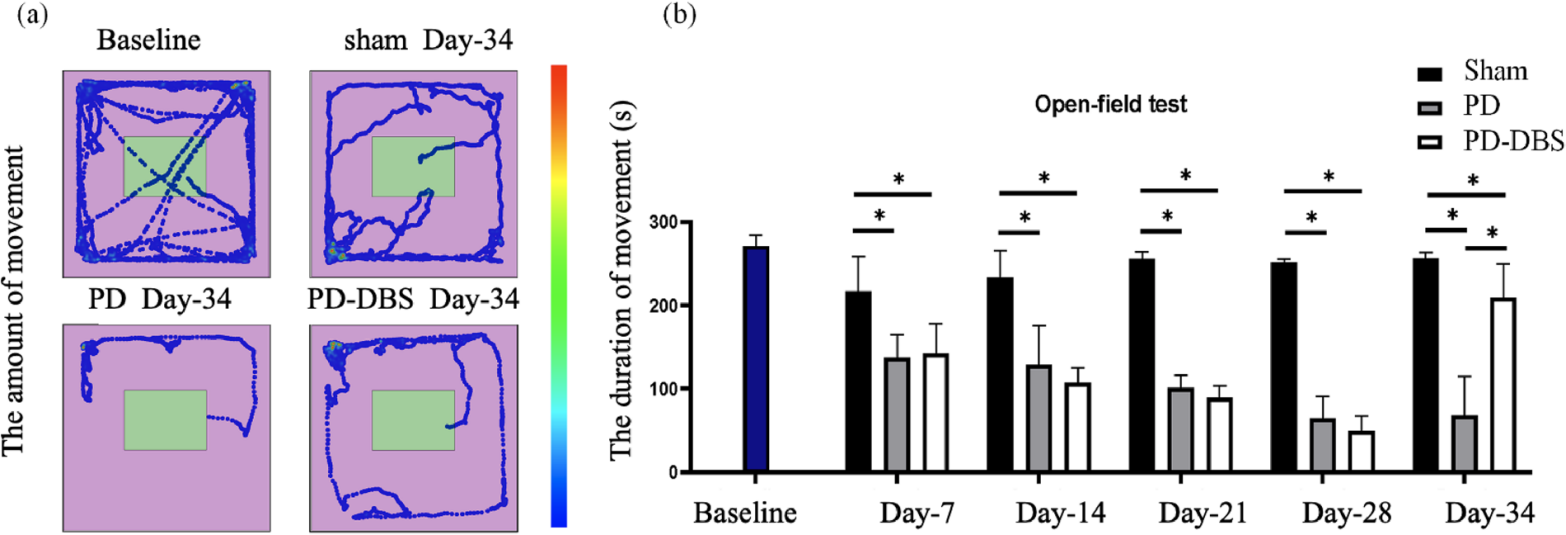
The open-field test verification. (**a**) The amount of movement. (**b**) The duration of movement. *P < 0.05.

For the spontaneous movement in open-field test, the differences between hemi-PD and sham control rats were further verified by the amount and duration of movement. Before drug injection, rats moved longer distances and for longer time, and explored central areas, which was consistent with the results of normal rats (Fig. 4a). For the first behavioral test after drug injection, sham rats showed a slight decrease in the amount and duration of movement due to the lesion experiment, however, obvious decrease of movement was observed in PD and PD-DBS rats, which suggested the gradual emergence of motor dysfunction (Fig. 4b). In Day-34, the movement condition of PD rats was significantly reduced, and spent more time in the corner. However, the movement of sham rats was slightly reduced than baseline, and still active in the central area in Day-34. Surprisingly, for PD-DBS rats in Day-34, although there was no activity in the central area, the movement in the peripheral area was significantly increased compared with the PD group, which meant the long-term closed-loop DBS could enhance the movement ability of PD rats. The mean values of the duration of movement among three groups were compared using one-way ANOVA analysis, the data were considered statistically significant with *P* < 0.05.

### Action potential waveforms (spikes) sorting

The Offline Sorter^TM^ software (Plexon Inc) offered different sorting methods, including the valley-seeking algorithms, template matching sorting, and k-means. Studies comparing these spike sorting methods showed that valley-seeking algorithm could yield the most accurate result if the data was particularly noisy^56^. Although some missed spikes would be left, the subsequent manual classification could improve the accuracy of classification, therefore the valley-seeking algorithm was used in this study. Studies have shown that the spike data obtained from the microwire array electrode implanted in layer V of M1 could be classified into putative pyramidal projection neurons (PNs) and interneurons (INs)^40,57^.

**Fig. 5:**
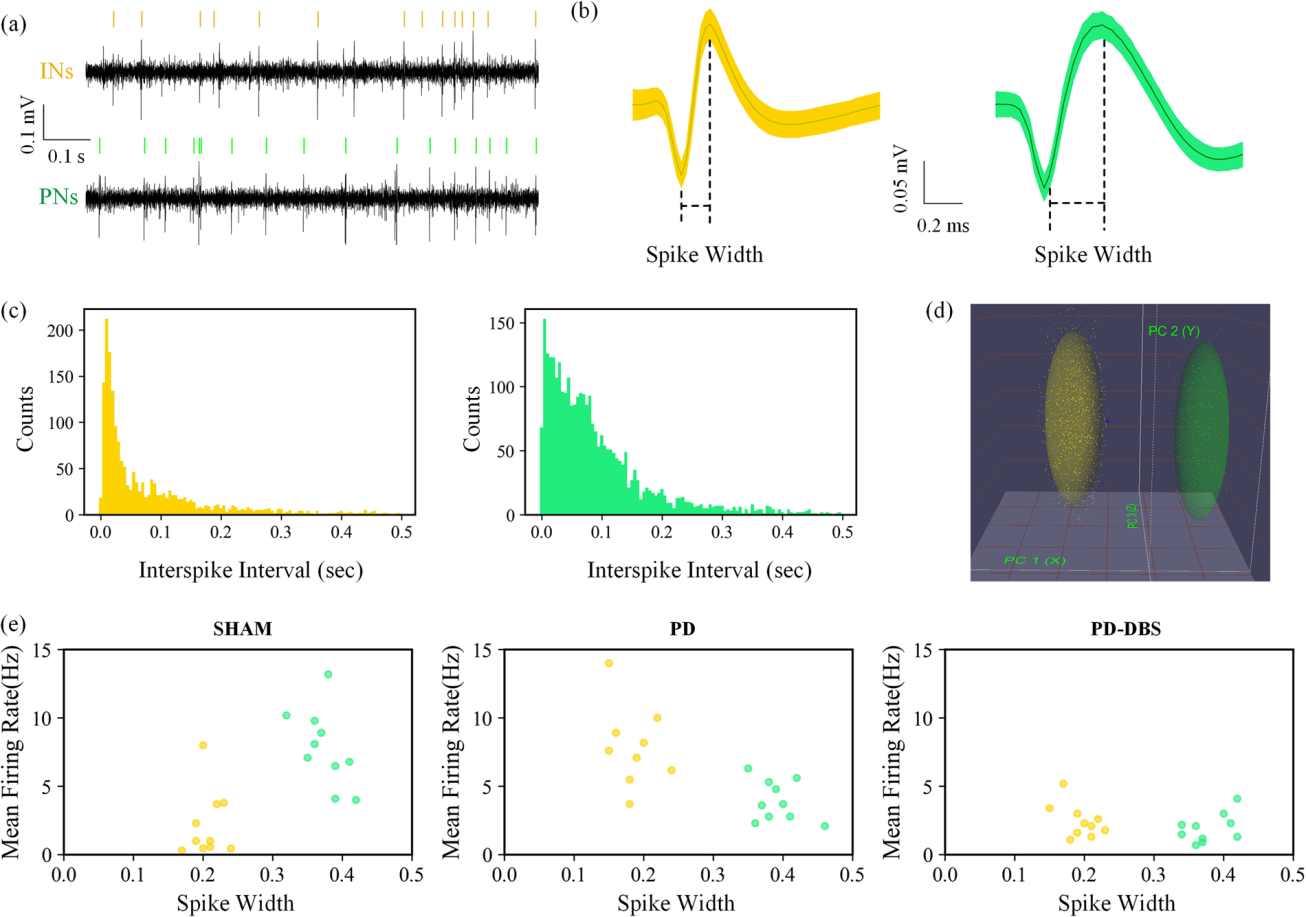
Example analyses of action potential waveforms sorting from M1-spike data. (**a**) The recorded spike data for two types of neurons. (**b**) The spike waveform of two types of neurons. (**c**) Histogram of interspike intervals of two types of neurons. (**d**) Neuron clustering based on principal component analysis in 3D space. (**e**) Clusters of putative PNs and INs based on spike width and mean firing rate features for the sham group, PD group, and PD-DBS group.

Figure 5a was the spike signal of these two neurons, compared with the putative PNs, the INs have shorter spike width (Fig. 5b). As shown in Fig. 5c, the number of putative PNs was greater than that of INs, and the distribution of interspike interval was less centralized than INs. These two types of neurons could be distinguished by the principal component analysis (Fig. 5d). Compared with the normal state, the spike amplitude was reduced under the PD state, and the spike width was basically unchanged. Compared with the PD state, closed-loop DBS had a greater modulation effect on putative INs than PNs, the spike amplitude of INs was larger than that of the PD state^58,59^. The sorted neurons were labeled into PNs and INs in the dataset, which could further be used to study the variation of spike rate and spike pattern under different states.

### Power spectral density of LFP signal

**Fig. 6:**
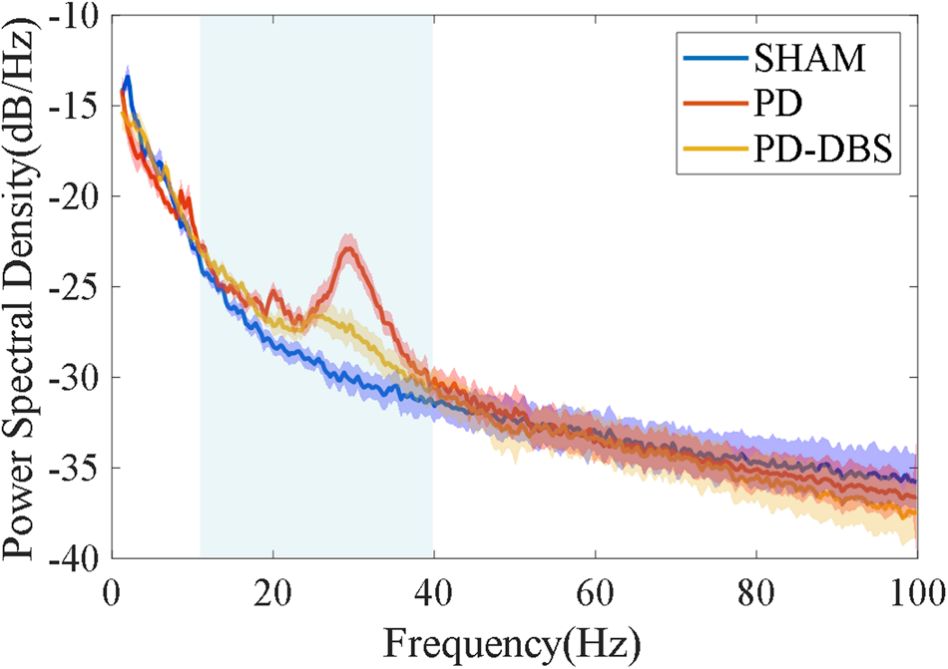
Power spectral density comparison among the sham group, PD group and PD-DBS group for the lesioned side, the light blue square represents the range of the beta band (12–40 Hz).

Here, we provided example spectral power analysis that could be performed using the LFP signal. First, the time points corresponding to the peak of each stimulus artifact in the wideband signal were identified by establishing an appropriate threshold line, and all sampling points affected by these artifacts were substituted with linear interpolation to remove stimulation artifacts for the PD-DBS group. Then, the clean LFP signal was obtained by fourth order Butterworth band-pass filtering (0.5–300 Hz) and 1000 Hz downsampling of the wideband signal, and the notch filtering was executed at 50 Hz and its harmonics to remove power line noise. The welch spectral power analysis method was used to analyze the clean LFP signal, with the window function selected as Hamming, the window length as 3 seconds (3000 sample points), and overlap rate as 50% via trial-and-error. As shown in Fig. 6, the PD group was determined by the large amplitude of oscillations in the beta band (12–40 Hz), while these oscillations were not obvious for the sham group, and the enhanced beta oscillations were suppressed for the PD-DBS group.

### Spectral power analysis of LFP signal under sleep, wake and walk states

For the rats’ states (sleep, wake, and walk), the difficulty lies in the judgement of the sleep state. According to current studies, both the rat and mice adopt a curled-up position with lowered heads when they enter the sleep state^60^. In addition, in the bright environments, rats tend to curled-up and sleep with their eyes closed, while in the dark environments, they tend to sleep with their eyes open. The sleep state was judged by the body curling states and the real-time recorded spectrogram of LFP signal. During the sleep state, the real-time spectrogram of LFP displayed by the PlexControl software (Plexon Inc) was mainly lower than 10 Hz^61^. Therefore, we implemented a dual-expert approach for the classification of rats’ states, with one expert visually judged the behavior of rats and another was concentrated on spectrogram of LFPs, then these two experts collaboratively reviewed their findings to achieve a consensus.

**Fig. 7:**
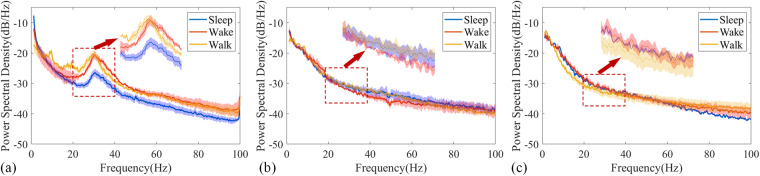
Example analyses of power spectral density under sleep, wake and walk states from bilateral M1-LFP data. Power spectral density of the (**a**) lesioned side and (**b**) contralateral side for PD group. (**c**) Power spectral density for the sham group. Each line represents the average of three episodes data collected on the same day, with the shaded portion representing their respective standard deviations.

The averaged the LFPs power spectral density analysis results of three episodes collected on the same day under the same state were shown in Fig. 7. On the lesioned side of PD group, the power spectral density in the beta range under the sleep state was lower than the walk state (Fig. 7a). However, there was no significant difference in the power spectral density between the contralateral side (Fig. 7b) of PD group and the sham group (Fig. 7c) under three states. In order to further explore the reasons for the existence of the aforementioned differences, we conducted spectral power analysis under different activity states for a single episode.

**Fig. 8:**
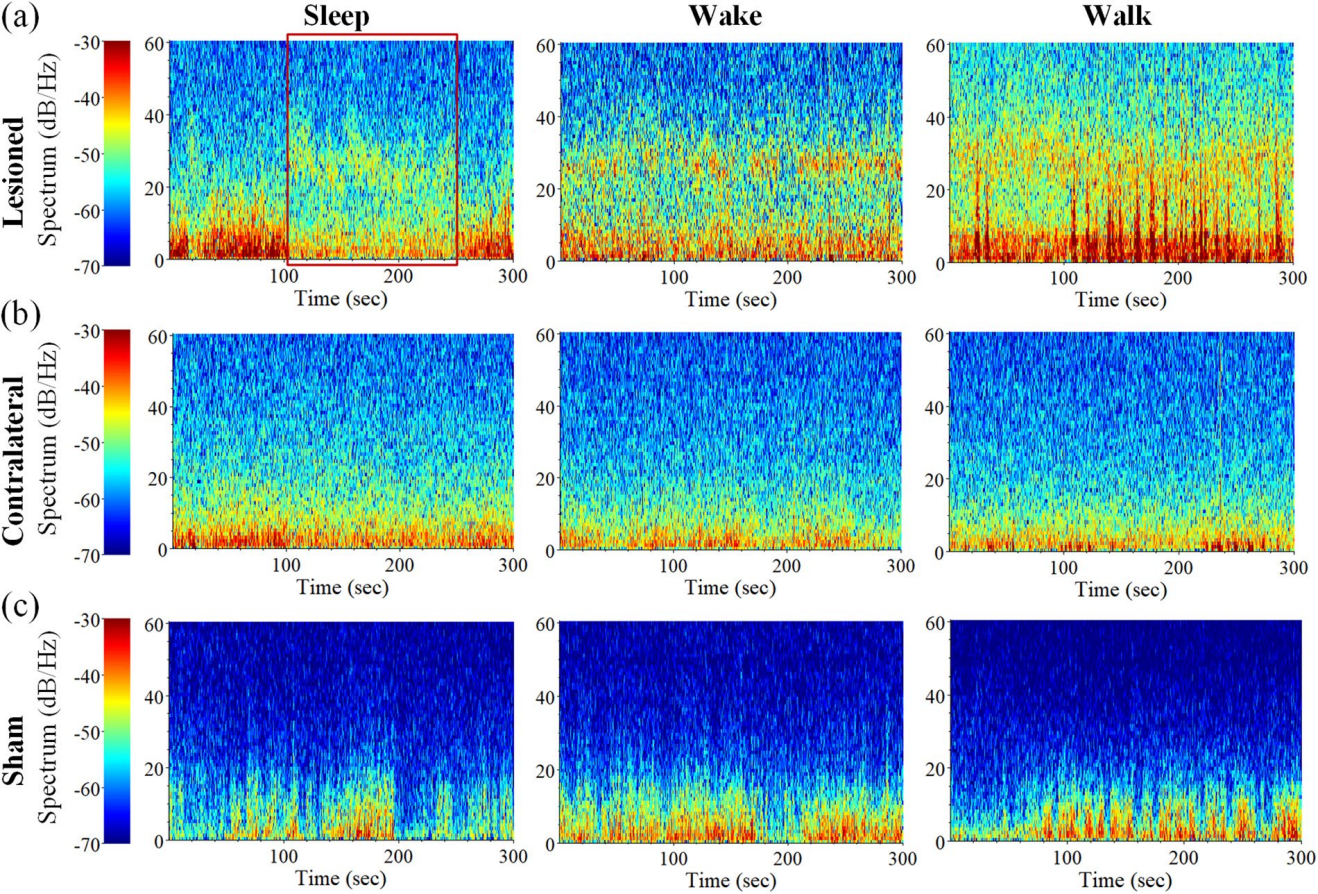
Example analyses of spectral power under sleep, wake and walk states from bilateral M1-LFP data. Spectral power of the (**a**) lesioned side and (**b**) contralateral side for PD group. (**c**) Spectral power for the sham group. The red square in (**a**) represents the observed abnormal increase of beta band oscillatory activity on the lesioned side during the sleep state.

Due to the inability of rats to maintain the same state for a long time, except for a few recording episodes, most of the electrophysiological dataset was with a collection time of 300 seconds. Figure 8a revealed a significant increase in beta band oscillation activity on the lesioned side during the walk state, while the corresponding beta band activity during the wake state was lower compared to the walk state. Surprisingly, during the sleep state, the rats could appear anomalous body movements occasionally such as twitching of limbs, the lesioned side also showed elevated beta band oscillation for some periods, labelled by the red dotted line in Fig. 8a. Besides, studies proved that during the rapid eyes movement (REM) sleep of PD patients, similar or even higher beta band oscillation activity than the wake state was recorded^54^. Thus, it could be inferred that PD rats also had high beta band oscillation activity during REM sleep, and similar findings were observed in the SNpr nucleus^62^. This also suggested that the cortico-basal ganglia-thalamic circuit was not only functionally connected anatomically, but also interconnected in terms of neural oscillatory activity. For the contralateral side of PD rats (Fig. 8b) and for sham rats (Fig. 8c), no obvious beta band activity was observed under all three states.

### Spectral power variation of LFP data for 5 days closed-loop DBS

**Fig. 9:**
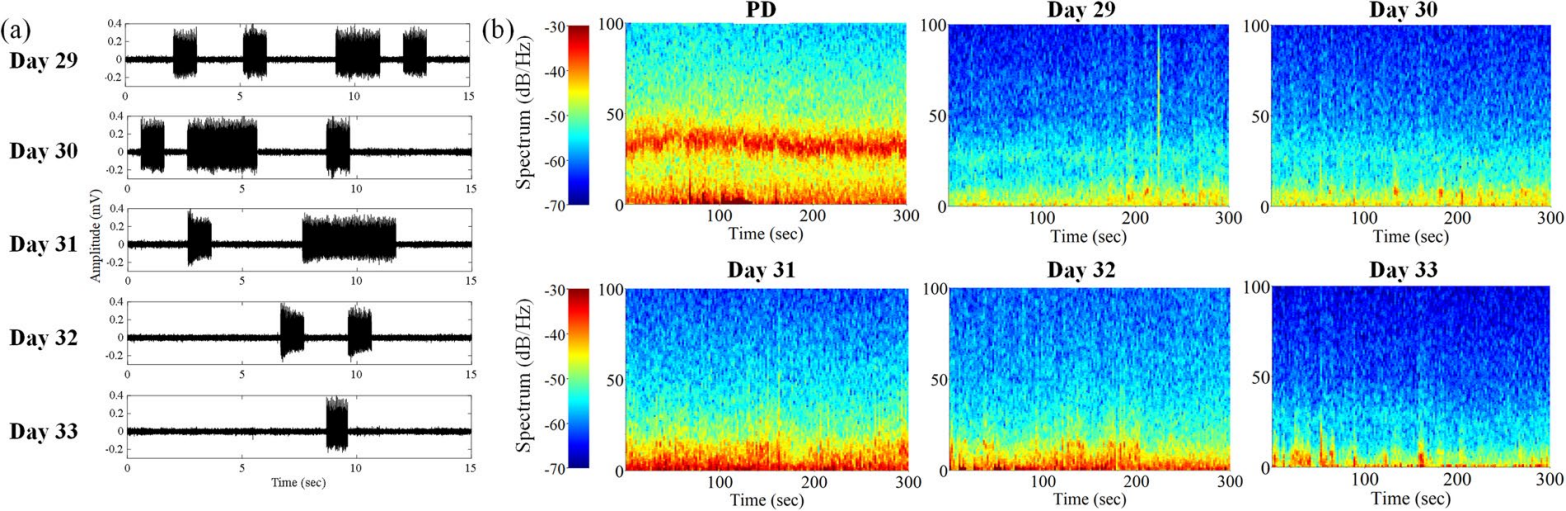
Example analyses of power spectral variation with 5 days closed-loop DBS at the same time period. The lesioned M1-LFP signal with DBS artifact for 5 days (**a**), and the corresponding power spectral variation for 5 days under walk state (**b**).

The closed-loop DBS was applied for 5 consecutive days (day 29 to day 33), and the corresponding spectral power of the M1-LFP signal in the lesioned side was calculated. The closed-loop DBS was performed based on the M1-LFP oscillation activity, Fig. 9a was the lesioned M1-LFP signal with stimulus artifacts, which indicated that the inter-stimulation-interval was prolonged and the duration of stimulation was relatively shortened as time went on. Figure 9b was the corresponding spectral power analyses of these 5 days, the application of closed-loop DBS suppressed the elevated beta band oscillation activity of PD state. Notably, although the applied stimulation was significantly reduced during day 33, a re-elevation of beta band activity was not detected.

Notably, the animal models constructed by injection of neurotoxin or gene knockout cannot fully mimic the clinical characteristics of PD. However, the existing animal models could simulate the characteristics of PD from different aspects, which are helpful for understanding the pathophysiology of PD and the mechanisms of DBS. Potential biomarkers discovered on animal models still need more studies to verify the safety and efficacy, and ultimately providing more stable and accurate feedback for characterizing different states of PD. In conclusion, results obtained from rodent models can further facilitate the optimization of DBS therapy and improve the life quality of PD patients.

## Data Availability

The code for stimulus artifact removal and power spectrum analysis is available on G-Node. (10.12751/g-node.lzvqb5)^55^.
